# Non-clinical studies required for new drug development - Part I: early *in silico* and *in vitro* studies, new target discovery and validation, proof of principles and robustness of animal studies

**DOI:** 10.1590/1414-431X20165644

**Published:** 2016-10-24

**Authors:** E.L. Andrade, A.F. Bento, J. Cavalli, S.K. Oliveira, C.S. Freitas, R. Marcon, R.C. Schwanke, J.M. Siqueira, J.B. Calixto

**Affiliations:** Centro de Inovação e Ensaios Pré-clínicos, Florianópolis, SC, Brasil

**Keywords:** Non-clinical drug discovery and development, Target discovery and validation, Proof of principles, Animal research robustness

## Abstract

This review presents a historical overview of drug discovery and the non-clinical stages of the drug development process, from initial target identification and validation, through *in silico* assays and high throughput screening (HTS), identification of leader molecules and their optimization, the selection of a candidate substance for clinical development, and the use of animal models during the early studies of proof-of-concept (or principle). This report also discusses the relevance of validated and predictive animal models selection, as well as the correct use of animal tests concerning the experimental design, execution and interpretation, which affect the reproducibility, quality and reliability of non-clinical studies necessary to translate to and support clinical studies. Collectively, improving these aspects will certainly contribute to the robustness of both scientific publications and the translation of new substances to clinical development.

## A brief history of drug development

The pharmaceutical industry, which involves an annual market of more than one trillion US dollars, has undergone remarkable progress regarding the development of new drugs. However, it is a relatively young industry of about 70 years.

Paul Ehrlich (1854–1915), a German physician researcher and Nobel Prize winner for Medicine and Physiology, created in 1908 the following concept: “*Corpora Non Agunt Nisi Fixata*”, meaning “substances do not act if they are not bound”. The still prevailing concept regarding drug mechanisms of action was primordial in the creation of synthetic drugs and the subsequent growth of large pharmaceutical corporations. Many decades later from this initial concept, which provided the first indication of molecular targets, pharmacological receptors were discovered, allowing the development of most drugs currently on the market. With these events, the term "chemotherapy" also appeared. It is important to note that Ehrlich was one of the first researchers to perform chemical synthesis in order to develop a drug to treat syphilis. Thus, in 1906, in partnership with Hoechst, the scientist decided to carry out structural changes in substances called arsenobenzenes. After synthesizing 606 substances, he discovered arsphenamine (also known as compound 606), later named Salvarsan, which was used for the treatment of syphilis. In further studies with compound 606, Ehrlich designed the first protocols for the evaluation of clinical efficacy, which were distributed to several clinics in Germany, establishing the beginning of clinical research (1,2).

In the beginning of the 20th century, the world did not have drugs developed based on scientific research concerning safety (toxicology) and efficacy. Drugs were mainly derived from plants, with few substances of synthetic origin. In 1906, the Food and Drug Administration (FDA) was established as the first agency to regulate the quality of food and medicine. However, at that time, the FDA was concerned only with limited aspects of drugs, such as their physical and chemical characteristics and their purity. Thus, drugs contaminated with heavy metals, such as mercury and arsenic, were often found. Substances such as morphine, cocaine, and many others that have been later banned in medicine, or that are currently under strict control, were freely marketed (3).

Based on a severe intoxication that occurred in the USA due to the use of a toxic solvent (73% diethylene glycol) used to solubilize sulpha drugs, Ceilling and Connon (4 [Bibr B05]) proposed the basic principles for the conduct of clinical studies that were approved and then incorporated into the Nuremberg Code in the following years. These principles have been guiding scientific studies for the development of new drugs until today. It was established that, prior to human administration, all new substances should have the following characteristics: well-established chemical composition, method of preparation and degree of purity; acute and prolonged toxicity tests assessed by repeated doses (safety) in different animal species; histopathology analysis in several animal organs, especially in kidneys and liver; known absorption and excretion mechanisms and drug tissue concentration; and known possible interactions with other substances and food.

From the Nuremberg Code landmark, the first multidisciplinary research groups arose, including physicians, pharmacologists, physiologists and chemists, which was the basis for the emergence of the large global pharmaceutical corporations. The first core of clinical research involving physicians and biostatisticians also emerged. These were the main factors responsible for the extraordinary impulse observed in the field of new drug development, which enabled major advances in medical practice between 1950 and 1980. Drugs were developed based on the identification of new therapeutic targets, namely pharmacological receptors, as proposed earlier by Ehrlich. This was possible due to enormous advances in the basic sciences between 1970 and 1980, mainly on cellular and molecular biology and medicinal chemistry. From this knowledge, new drugs could be developed against specific targets, and diseases of high prevalence, such as hypertension, schizophrenia, depression, hyperdyslipidemia, diabetes, cancer, and others, could be better controlled. Therefore, a substantial increase of life expectancy and quality of the world’s population was observed (for reviews, see 3,5).

## Early studies of drug discovery

The drug discovery industry typically follows one of two models to identify hits and lead compounds: the phenotype (or physiology) or the target-based approach, which differ in the way that they lead to therapeutic target identification and small compounds selection/optimization (6–8). Therapeutic target is a broad term that indicates the site where the substance will bind/act promoting its biological activity. In the phenotype screening approach (“forward pharmacology” or “classical pharmacology”), substances are characterized in accordance to their physiological effects based on disease-relevant assays (e.g., cell-based phenotypic assay, isolated tissue or animal models of disease) developed using basic biological and disease knowledge or clinically effective drugs. This approach can potentially lead to an identification of a molecule that modifies the disease phenotype through interaction or modulation of a previously undefined target (receptors, transcription factors, cytoskeleton proteins, enzymes and others) or simultaneously on multiple targets (6,9). The phenotypic screening approach requires additional effort to discover the target of small molecules, since that direct interaction with a single target is not always responsible for phenotypic observations. Therefore, many drugs exhibit side effects owing to interactions with ‘off-target’ proteins (10,11), and even small molecule-induced phenotypes observed in cell culture may represent the superposition of effects on multiple targets (12,13).

In the target-based approach (also know as “reverse pharmacology” or “reverse chemical biology”), the goal is to develop drugs that affect the specific and known target, which are mostly proteins (receptors, enzymes, transporters, ionic channels, intracellular receptors, etc.) previously discovered and linked to the human disease. The early steps of the target-based approach consist in target identification and validation. The drug design process occurs frequently, but not necessarily, by means of computational modeling approaches that are able to selectively interact with the identified target. The main advantage of target-based model is the high screening capacity, which allows the implementation of rational drug design and accelerate the preclinical drug development; however, the molecules can only be optimized against a small number of targets simultaneously. Other advantages of the target-based approach compared with phenotype screening are the direct structure-activity relationship (SAR) optimization and the knowledge of the mechanism of action. In both cases (target- or phenotype-based model), it is necessary to validate the disease model for proof-of-principle studies (6,7,9).

Advances in molecular biology, genomics and proteomics led to the replacement of the phenotypic assays by screens against defined targets implicated in disease. This approach has delivered many clinical candidates, but several potential drugs failed in phase II and III clinical studies mainly by invalidated targets. Target-based screening is likely to provide very good drug candidates for monogenic diseases. However, most of the more prevalent human diseases such as Alzheimer’s disease, asthma, diabetes and other complex diseases are most likely multifactorial and therefore drugs would require interaction with multiple targets to produce clinically meaningful effects. In addition, drugs with high potency and with selective interaction with a single target may increase the risk of adverse events or be limited by redundancies and adaptive resistance (14). For this reason, phenotypic screening has emerged as a renewed approach in drug discovery, as some researchers have concluded that reductionist approaches such as target-based screening are useful, but may also limit the breadth of new findings. Although some companies are embracing this revival, others remain focused on target-based screening (15).

The cost of the benefit of phenotypic-based screennig is that the precise protein targets or mechanisms of action responsible for the observed phenotypes remain to be determined. This process has historically been slow and at times unproductive. Then, the challenge of searching the target with the phenotypic screening has discouraged some drug developers from using this approach in their early discovery stage. Additional technologies that are emerging, such as the combination of genetics and chemical proteomics (15), have caused target identification to be more feasible. On the other hand, knowledge of the target for a drug candidate is not necessarily an absolute requirement during initial development stages. However, the definition of the on- and off-target space remains an important step toward full development; it would facilitate optimization of the drug candidate (16).

### Identification and validation of therapeutic targets

The targets on target-based drug discovery are identified using several molecular tools and strategies that include the evaluation of DNA/RNA (genomic) and proteins (proteomic) that have been correlated with a human disease. Integral use of molecular tools such as nucleic acid microarray, protein microarray, tissue and cell microarray, antisense oligonucleotides, RNA interference, zinc finger proteins, among others (7,17) can identify the target related with the human pathology in question. On the phenotypic-based approach, the substances activity is previously observed and then the target is identified. For this reason, efforts have been made to clarify the target or targets responsible for the phenotypic effects after obtaining the lead substances. The process of target identification in phenotypic-based approach is also known as deconvolution. Target deconvolution can be obtained by chemical proteomic-based approaches (affinity chromatography, activity-based protein profiling, label-free techniques), expression cloning techniques, *in silico* approach and others (7,18).

After identification, the therapeutic target should be validated. The aim of the validation is to evaluate whether the modulation of the therapeutic target is able to generate a reasonable biological response (19). Validation techniques range from *in vitro* tools to the use of whole animal models, and to modulation of a desired target in diseased patients (20). Nevertheless, target validation is not a one-step experiment, but an ongoing part of a strategy program, which begins with target identification and is not complete until the definitive clinical study (21). The most accepted criteria for target validation during drug discovery are based on three categories: 1) demonstration of the target protein expression or mRNA in relevant cell types or in the target tissues from animal models or patients, 2) demonstration that modulation of the target in cell systems results in the desired functional effect, and 3) demonstration that the target has a causal role in producing the disease phenotype in animal models and/or patients (22).

In most situations, the initial steps of therapeutic target validation are obtained using *in vivo* or *in vitro* assays and involve protein or messenger RNA expression in human samples by using immunohistochemistry and *in situ* hybridization techniques, respectively. Although protein characterization is the favorite option, this technique could be limited by the unavailability of specific antibodies to a specific target (23). However, the association of the target protein with diseased or target tissue is rarely considered sufficient for target validation. The functional association of the target with disease modification is also required. Furthermore, it is also possible to explore the target validation in transgenic and gene knockout animals, using small molecule inhibitors, antisense oligonucleotides, and small interfering RNA (siRNA) (24). However, it is important to highlight that animal models often do not depict the full disease phenotype or share the same pathophysiology as observed in patients. Frequently, the targets in the animal models may have a different tissue expression and distribution when compared to humans. Also, the pathophysiological pathways in patients could be evolutionarily diverged from the animal models and serve a different mechanism of action. Therefore, to avoid all the above mentioned issues it is most desirable to validate a target in at least two species with different approaches to gain further confidence in clinical translatability before entering the intensive clinical phase of drug development (21).

### *In silico* assays

The term ‘*in silico*’ is used to define experimentation performed by computers and is related to the more commonly known biological terms *in vivo* and *in vitro.* It defines the use of information in the creation of computational models or simulations that can be used to make predictions, suggest hypotheses, and ultimately provide discoveries or advances in medicine and therapeutics.

The advantage of *in silico* studies is the speed of execution, the low cost and the ability to reduce the use of animals. The use of *in silico* methods have been an interesting strategy to accelerate the discovery of potential new drugs. The design of *in silico* drugs prototypes ranges from the study of the structure-activity relationship until toxicology and pharmacokinetic studies (ADME: absorption, distribution, metabolism, and excretion) (25).

Regarding *in silico* pharmacodynamics, homology modeling is based on the homology between amino acid sequences, which provides information about the structural and functional similarities. This methodology is used to map the therapeutic target structures and cover their three-dimensional structures (25). Another important methodology frequently used for pharmacodynamics evaluation is molecular docking, which consists in predicting the bioactive conformation of a small molecule (ligand) in a binding site of a macromolecule (target protein). This method provides a good approximation of the expected conformation and orientation of the ligand in the protein cavity and then predict the associated binding affinity (26).

The knowledge of macromolecular targets or structures like ligand-receptor complex, allows the use of drug design strategies based on receptor structure. In contrast, when the structure of the target is not known, methods of drug design based on the structure of the ligand may be used, investigating the properties and characteristics of bioactive ligands series. The integration of experimental and computational methods have a great importance in identifying and developing new drugs from collections of real or virtual compounds.

Virtual screening is based on ligand structure (ligand-based virtual screening) according to the information about the topological arrangement of biological targets, using as a prerequisite the detailed 3-dimensional macromolecule data. This information may be obtained by analysis of crystal structures, NMR or homology modeling (27). Virtual screening based on receptor structure (target-based virtual screening) employs methods of molecular docking on the analysis of large compound databases in order to characterize an ideal chemical and biological space and allowing selection of compounds for biochemical and/or biological tests (27).

*In silico* modeling of ADME proprieties has been performed using different approaches. These methods range from database approaches such as quantitative structure-activity relationship (QSAR), similarity searches, and three-dimensional QSAR, to structure-based methods such as ligand-protein docking and pharmacophore modeling (28,29). Indeed, parameters that could be observed by QSAR could also anticipate solubility data, membrane permeability, volume of distribution, plasmatic protein binding, cytochrome P450 (CYP450) enzymes interactions, which are important for substance metabolization, etc. (30). Thus, *in silico* studies are extremely important to adequately guide new drug development, from its conception to the performance of *in vitro* and *in vivo* studies.

### High-throughput screening assay

For about two decades, the identification process of new substances with activity on a specific target was slow, difficult and with limited yield. The advent of genomic sciences, rapid DNA sequencing, combinatorial chemistry, cell-based assays and automated high-throughput screening (HTS) has led to a “new” concept of drug discovery.

Currently, HTS is a well-established process in lead discovery for pharma and biotech companies and is now being set up also for basic and applied research in academia and hospitals (31 [Bibr B32]). HTS operations are highly automated and computerized to handle sample preparation and assay procedures, so a large number of hypothetical targets can be incorporated into molecular or cell-based assays and exposed to many compounds representing numerous variations on a few chemical classes, which can certainly accelerate the generation of lead substances (32–34).

During the rise of HTS in the early 1990s, 96-well microplates were the initial format for sorting and handling of chemical substances in most pharmaceutical and biotechnology companies. However, in the last decade there has been great effort to develop different types of plates that accommodate a larger number of samples, simultaneously. Thus, 384-well microplates arose, which accommodate four times more samples than the 96-well microplates. Most biochemical or cell-based assays can be adapted to a 384-well microplate without any problem and this plate format has been established as the standard for the storage of substances and screening assays in many pharmaceutical and biotechnology companies (31,35). Improvements in instrumentation have led to miniaturization and to the 1536-well format plates, with reaction volumes between 2 and 10 µL. This format may be used for some assays (36).

There are several classes of molecular targets in HTS assays. Enzymes such as kinases, proteases, phosphatases, oxidoreductases, phosphodiesterases, and transferases, among others, comprise the majority of biochemical targets in today’s new molecules’ discovery (37,38). Among cell-based targets, many GPCRs (G-protein coupled receptors), nuclear hormone receptors and some types of ion channels are very well suited for the screening of large molecule collections with today’s screening technologies.

Among the different types of assays used in HTS, the biochemical and cell-based assays are the most important. The main objective of cell-free assays for HTS is to minimize the number of steps required in setting up the assay and detecting the activity. Biochemical assays are cell-free *in vitro* systems that involve enzyme/substrate reactions, receptor binding or protein-protein interactions and other more complex models, as *in vitro* transcription, and give the direct information on the nature of the molecular interaction (34,39).

Cell-based assays are an attractive alternative to biochemical cell-free assays for HTS. Cell-based assays, using the function of the target protein, mimic the biological role of the protein in a specific disease state more closely than cell-free assays (40). Cell-based assays have pronounced advantage in comparison to biochemical assays because they involve the evaluation of parameters such as cell growth and proliferation, membrane transport, metabolism, cytotoxicity, signal transduction pathways, reporter gene, agonists and antagonists identification, etc. (34).

It has been proposed that HTS is a contributory factor responsible for the decline in productivity in the pharmaceutical industry. Moreover, some experts believe that this technique could reduce the creativity demanded by the drug discovery process. This observation has caused discussions about the real value of the HTS method to the discovery of new drugs (41–43). However, HTS has matured to become an integral part of pharmaceutical research and a cornerstone in the expansion of biomedical knowledge. HTS has provided an important tool to enhance basic scientific research and to help in the discovery of new drugs, enabling advances in the development of pharmaceutical products. Furthermore, many benefits have arisen from the development and implementation of HTS technologies. It must be emphasized that all strategies of identification have strengths and weaknesses, and it is their wise combination that can lead to the most successful strategy in modern drug discovery (44).

## Proof-of-concept (or principle) and study of the mechanism of action of a substance

The development of a new drug requires a meticulous and strategic scheme of different evaluations that encompass a chain of events that may overlap and transcend the non-clinical and clinical investigation steps. The evaluation of non-clinical activity (efficacy) of a new drug candidate includes *in vitro, ex vivo* and *in vivo* assays that can be carried out throughout all stages of drug development. These tests are essential to provide the basic knowledge about the drug pharmacodynamics and are required for later entering into clinical studies.

Due to improvements in the basic sciences of the biomedical field, it is natural that the methods for analyzing the non-clinical efficacy of a drug candidate are thus constantly advancing. Although it is currently essential to use experimental animals, efforts have been made to reduce the use of *in vivo* techniques to a minimum. In fact, the rational use of animals has been discussed for several years, especially with the emergence of guidelines from the National Center for the Replacement, Refinement and Reduction of Animals in Research (NC3Rs) in 2004 and, more recently, the ARRIVE (from Animal Research: Reporting of *In Vivo* Experiments) guideline (45,46), which describe several important steps in the planning and execution of non-clinical efficacy tests, such as proper experimental design, powerful analysis techniques and thorough evaluation of the results. Despite efforts for the standardization and validation of alternative methods, *in vitro* assays for the evaluation of non-clinical efficacy have major limitations and the use of experimental animals in the process of drug development is still essential to meet the standards required by the main agencies that control the registration and use of drugs.

### *In vitro* and *ex vivo* assays

Usually, *in vitro* assays are performed in the early stages of the drug discovery process, when the selectivity and possible interactions of the candidate substance towards the desired therapeutic target are established. At this step of drug development, several molecules, previously selected by *in silico* methods and/or by HTS screening, can be tested (47). After the selection of promising substances, one may conduct further *in vitro* tests for the initial evaluation of biological activity. For this purpose, a variety of assays using lineage or primary culture cells of human or murine origin are now available. Through these *in vitro* assays, it is possible to observe the activity of the substance upon different features, such as the induction of cell death and proliferation, changes in gene expression, changes in the protein profile, biochemical dosage of mediators, changes in cell cycle assessment, multidrug resistance potential, and others (48–51). However, it is important to state that *in vitro* evaluations carried out in cells or in an individual target, such as an enzyme, can eventually provide false results. For this reason, the candidate drug should also be evaluated by *ex vivo* models, in which more complex structures are considered. In these models, a small number of animals are used for biological material harvest, for example, smooth muscle of the gastrointestinal tract, airways, urinary tract, blood vessels, brain, cardiac muscle, endocrine glands, liver, spleen, among others (52).

The monolayer, also known as two-dimensional (2-D) cell culture, is an important tool to *in vitro* drug discovery and development, but it has several disadvantages when compared with feasible three-dimensional *in vitro* cell culture. The 3-D cell culture is complex and maintains several functions of the native tissue and, in some cases, the physiological response to drugs. New technologies have advanced and recently 3-D printing of tissue and organs has been developed with characteristics and function similar to human organs. The bioprinting possess several advantages such as tailored microarchitecture, high-throughput capability, coculture ability, and low risk of cross-contamination. Furthermore, it offers very precise spatial and temporal control on placement of cells, proteins, DNA, drugs, growth factors, and other bioactive substances to better guide tissue formation (53, http://www.ncbi.nlm.nih.gov/pubmed/?term=Peng%20W%5BAuthor%5D&cauthor=true&cauthor_uid=27296078). This new and powerful technology for drug discovery and development is a promising method for the development of organ-on-a-chip models, which mimic several functions of physiological tissue. Some of these models are under development, such as lung, kidney, artery, hearth-on-a-chip, or are integrating several organs named human-on-a-chip. Bioprinting is a promising method for advancement in tissue fabrication towards physiologically relevant tissue constructs and organoids for pharmaceutics, drug testing, and HTS (53,54 [Bibr B55] [Bibr B56] [Bibr B57]).

### *In vivo* assays

Based on the initial results of *in vitro* and *ex vivo* studies, as well as on information about the therapeutic target, the clinical indication and the knowledge of the pharmacokinetic profile of the candidate substance, it is possible to outline *in vivo* tests with the aim of determining the efficacy in a certain biological model. Furthermore, the selection of which *in vivo* experiments will be conducted should be discussed on a case-by-case basis and the most powerful and scientifically relevant methods should be chosen. Generally, disease models can be divided into three types: i) those which are induced by an invasive procedure (physiological), ii) those which are induced by a substance (pharmacological), and iii) those using genetically modified animals (genetic) (for examples of disease models, see 55-57). All of these models are designed to develop an abnormality similar to which occurs in the disease under investigation. Moreover, the *in vivo* models can be subdivided into acute or chronic, depending on the duration of disease.

Although animal models might generate relevant information on the non-clinical efficacy of a substance, they are far from reproducing all of the signs and symptoms of human diseases, and definitive proof of the efficacy can only be confirmed after the completion of phase II clinical trials. Nevertheless, animal tests are essential to guide the early stages of development, particularly for making decisions regarding whether to continue the project (go/no-go decision).

A further important aspect to be noted when efficacy evaluation of a substance in non-clinical *in vivo* studies is planned is the confirmation that the therapeutic target that has been identified and validated as a human protein has similarities with an animal protein. Normally, the proof of principle assays (also referred to as proof of concept) are carried out in species such as *Mus musculus* or *Rattus norvegicus* and, if there is no correlation with the human protein (therapeutic target), the animal experiments will not generate reliable results. The use of different animal species during the development of a pharmaceutical product is one of the main reasons for failure in this process, due to the differences between species and difficulties in translating the results to humans. Indeed, the pathophysiology of certain diseases is frequently distinct between species (58,59). Moreover, the metabolism of animals and humans are often distinct, which may result in changes in the duration of the test substance activity, affecting both the pharmacology as well as the toxicology, generating inconclusive results (60).

### Mechanism of action assessment

The mechanism of action of a new substance is defined as the set of target and effector proteins necessary to produce its pharmacological effect in a specific cellular context (61). When the drug discovery process of a small molecule follows the target-based approach, the next steps regarding efficacy testing and evaluation of the mechanism of action become far more simple. However, when the drug discovery procedures follow phenotypic-based screening and there is no direct evidence about the therapeutic target, the mechanism of action can then be determined during the drug development, even when the candidate drug is already being evaluated in clinical trials. Moreover, there is a possibility that the drug mechanism of action may not be fully clarified but the drug may be approved for marketing. Indeed, the FDA has approved many drugs with unknown mechanism of action or target identification (62).

The use of the computational approach or molecular assays are possibilities for elucidating the mechanism of action of a new small drug (63,64 [Bibr B65] [Bibr B66] [Bibr B67]). In the computational approach, a rich set of data is necessary about the thousands of substances whose activities will be compared with drug candidates; the methods are mostly designed to assess the mechanism of action similarities or specific substance/target interactions (61,). Molecular assays rely on detailed three-dimensional structures of both substance and target proteins or on prior literature or database knowledge of mechanism of action of related substances (61). Furthermore, the molecular assays that can be used to elucidate the mechanism of action include affinity chromatography, activity-based protein profiling, label-free techniques, expression cloning techniques and others (7,18).

Much of the molecular approaches for elucidating the mechanism of action of a new small drug rely on direct binding assays (68–70). The binding assays are generally limited to the identification of high-affinity binding targets, rather than of all proteins responsible for substance activity. Then, the issue that arises with the application of this method is that indirect effectors, lower-affinity targets responsible for both desirable and undesirable pharmacological properties, and effects from tissue specific interactions and signals are neglected (61).

## Reproducibility, quality and reliability of non-clinical studies

A factor that has been attracting the attention of experts from the pharmaceutical industry and the scientific community in particular, in the last five years, relates to the robustness of non-clinical studies, even of those published in high impact international scientific journals, such as Nature, Cell, and Science, among others. Studies conducted by researchers from Amgen and Bayer Healthcare showed a very low rate of reproducibility (89 and 78%, respectively) of articles published in international journals (71,72). In addition, other groups assessed a greater number of non-clinical studies and confirmed the lack of reproducibility, ranging from 51 to 54% (73–76). These findings have caused a radical change in submission and evaluation rules for scientific papers in major specialized journals. Some critical points should be considered to eliminate any potential deviations that may interfere with and/or produce false results and affect the reliability, quality and reproducibility of the results of non-clinical studies, such as: i) sanitary quality of experimental animals; ii) animal distribution in the different experimental groups; iii) sample size calculation; iv) management of experiments; v) appropriate statistical analysis; vi) correct data handling; vii) quality of the reagents; and viii) need for negative and positive control groups (77–79). In order to reach high quality in non-clinical studies, it is imperative that the institution works in accordance with the institutional Good Scientific Practice, which is analogous to the Good Laboratory Practice (GLP). The GLP principles are a quality program related to organizational processes and experimental conditions, required for a non-clinical study to be accepted in the main international regulatory agencies (80). More recently, guidelines of a document named ARRIVE (Animals in Research: Reporting *In Vivo* Experiments) were developed, in consultation with the scientific community, as part of an NC3Rs initiative to improve the standard of reporting research using animals. It is a 20-item checklist, containing key information necessary to describe a study comprehensively and transparently. To date, these guidelines have been endorsed by over 400 scientific journals as well as by funding agencies, universities, and learned societies (http://www.nc3rs.org.uk/), in the hope that they will not only improve the quality of scientific reporting but also the internal validity of the research.

## Conclusion

In this review, early studies for the identification and validation of therapeutic targets and the *in silico* and high-throughput screening approaches were briefly discussed, as well as the proof of concept (or principle) assays that contribute to lead drug candidate selection which will advance the development process. Also reviewed were the efforts to reduce, or even banish, the use of experimental animals during the process of new drug development, which resulted in the expansion and validation of several alternative methods that are being adopted and recommended by the main international regulatory agencies. Furthermore, this review highlighted the current concern in relation to the reproducibility, reliability and quality of data, which affects the incidence of bias in non-clinical studies. Improvement of those aspects will certainly contribute to the robustness of both scientific publications and the translation of new substances to clinical development.
